# Access to Healthcare for Cancer Patients in Lithuania During the COVID-19 Pandemic

**DOI:** 10.15388/Amed.2021.28.2.9

**Published:** 2021-08-17

**Authors:** Vidas Petrauskas, Šarūnas Narbutas, Neringa Čiakienė, Guoda Gudelytė, Audrius Dulskas

**Affiliations:** Faculty of Medicine, Vilnius University, 29 Ciurlionio Str., LT-08406 Vilnius, Lithuania; Faculty of Law, Vilnius University, Saulėtekio al. 9, LT-10222 Vilnius, Lithuania Lithuanian Cancer Patient Coalition (POLA), Kalvarijų st. 235, LT-08311 Vilnius, Lithuania; Lithuanian Cancer Patient Coalition (POLA), Kalvarijų st. 235, LT-08311 Vilnius, Lithuania; Lithuanian Cancer Patient Coalition (POLA), Kalvarijų st. 235, LT-08311 Vilnius, Lithuania; Faculty of Medicine, Vilnius University, 29 Ciurlionio Str., LT-08406 Vilnius, Lithuania Department of Abdominal and General Surgery and Oncology, National Cancer Institute

## Dear Editor,

In December 2019 a novel beta-coronavirus (SARS-CoV-2), causing atypical pneumonia, was identified in Wuhan, China. The disease was called COVID-19 and rapidly spread across the globe. World Health Organisation (WHO) declared COVID-19 a pandemic [1] and special measures were imposed to stop the virus transmission such as avoiding crowded places, wearing personal protection equipment (PPE) when entering hospital, washing hands, reducing contact with people positive for COVID-19 symptoms and social distancing [2]. Despite all the efforts, COVID-19 caused an unprecedented healthcare crisis demanding quick response to current situation and service reprioritization [3]. 

Cancer patient care is of special concern during the COVID-19 pandemic. Oncology patients are more susceptible to COVID-19 and severe complications because of advanced age, immunosuppression caused by malignancy itself or anticancer therapy and comorbidities. A metaanalysis of 181.323 patients including 23.736 cancer patients from 26 studies showed that cancer patients infected with SARS-CoV-2 have a higher risk of death (odds ratio, OR 2.54) [4]. The same study found that cancer patients were a decade older than the normal population and have a higher proportion of comorbidities. Oncologists need to weight the potential risk for patients to be infected if they come to a healthcare facility and the possible damage of delayed inpatient cancer treatment [5]. If the treatment is on the way, one of the biggest concerns is that patients may suffer from serious side effects of chemotherapy and be unable to receive professional help on time because of healthcare facilities’ overload. 

During the quarantine, cancer patients have limited access to healthcare facilities. Despite the fact, that emergency service must be continued without disruption, there are still many cancer patients who are waiting for diagnostic tests or their treatment (radiotherapy or chemotherapy) had already began. 

**Figure 1.**
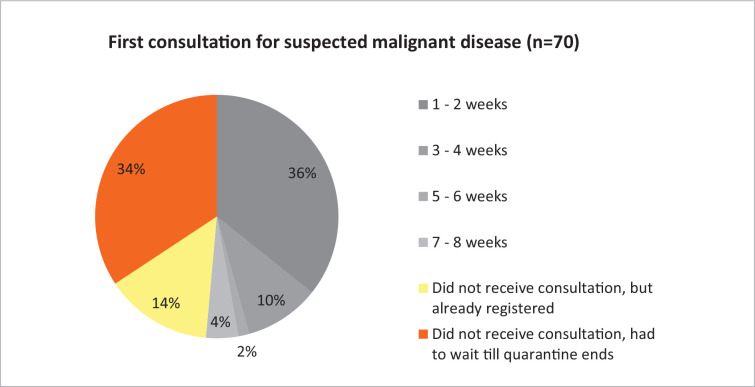
Time until the first consultation for suspected malignant disease

To find out how many patients did not receive necessary cancer care, Lithuanian Cancer Patient Coalition (*lith. POLA – Pagalbos onkologiniams ligoniams asociacija*) conducted a flash survey. This organization is the biggest umbrella patient organization in the country, uniting 31 nongovernmental organizations working in the cancer field and representing the interests of cancer patients, and aims to ensure that both patients and their families have access to effective treatment and the best possible informative, social, psychological and mental support. The POLA community flash survey on healthcare access during the COVID-19 pandemic was conducted from May 21st to June 1st. The survey was sent by an e-mail. In total, 670 people responded to the survey and 92.5% of them were cancer patients (Table 1). Almost three quarters of respondents sought medical care during the quarantine, and 34.3% of patients with suspected malignancy were told to wait for the first consultation until the end of quarantine (Figure 1). 

**Table 1.** Results of the survey of cancer patients and the treatment or surveillance during the COVID pandemics in Lithuania.

Questions	Answers
Gender (n = 670)	
Male	86 (13%)
Female	585 (87%)
Age (n = 670)	
18 - 25 y.	0.6% (n = 4)
26 - 39 y.	9.2% (n = 62)
40 - 55 y.	46% (n = 306)
56 - 65 y.	30% (n = 204)
> 65 y.	14% (n = 94)
Role (n = 670)	
Cancer patient	92.5% (n = 621)
Relative	5.5% (n = 37)
Other	1.8% (n = 12)
Stage of the disease (n = 670)	
Premalignant state	1.0% (n = 7)
Stage I	24.1% (n = 162)
Stage II	28.0% (n = 188)
Stage III	22.8% (n = 153)
Stage IV	12.7% (n = 85)
Not identified	3.1% (n = 21)
Other	7.6% (n = 51)
Did you seek medical service during the quarantine (n = 670)	
Yes	74.5% (n = 499)
No	25.5% (n = 171)
Received consultation within (n = 214)	
1 - 2 weeks	44% (n = 94)
3 - 4 weeks	10% (n = 22)
5 - 6 weeks	4% (n = 8)
7 - 8 weeks	5% (n = 11)
Did not receive consultation, but already registered	7% (n = 14)
Did not receive consultation, had to wait until the end of quarantine	30% (n = 65)
Follow up visit within (n = 357)	
1 - 2 weeks	25% (n = 90)
3 - 4 weeks	9% (n = 31)
5 - 6 weeks	5% (n = 16)
7 - 8 weeks	8% (n = 30)
Did not receive consultation, but already registered	14% (n = 50)
Did not receive consultation, had to wait until the end of quarantine	40% (n = 142)
Underwent diagnostic tests, radiology (n = 213)	
1 - 2 weeks	35.9% (n = 69)
3 - 4 weeks	13.5% (n = 26)
5 - 6 weeks	4.2% (n = 8)
7 - 8 weeks	6.8% (n = 13)
Did not receive consultation, but already registered	9.9% (n = 19)
Did not receive consultation, had to wait until the end of quarantine	29.7% (n = 57)
Underwent diagnostic tests, other (ultrasound, endoscopy) (n = 193)	
1 - 2 weeks	
3 - 4 weeks	31.6% (n = 61)
5 - 6 weeks	9.3% (n = 18)
7 - 8 weeks	5.7% (n = 11)
Did not receive consultation, but already registered	3.1% (n = 6)
Did not receive consultation, had to wait until the end of quarantine	8.8% (n = 17)
	41.5% (n = 80)
Underwent diagnostic tests, blood (n = 282)	
1 - 2 weeks	54.6% (n = 154)
3 - 4 weeks	6% (n = 17)
5 - 6 weeks	2.1% (n = 6)
7 - 8 weeks	2.5% (n = 7)
Did not receive consultation, but already registered	6% (n = 17)
Did not receive consultation, had to wait until the end of quarantine	28.7% (n = 81)

**Figure 2.**
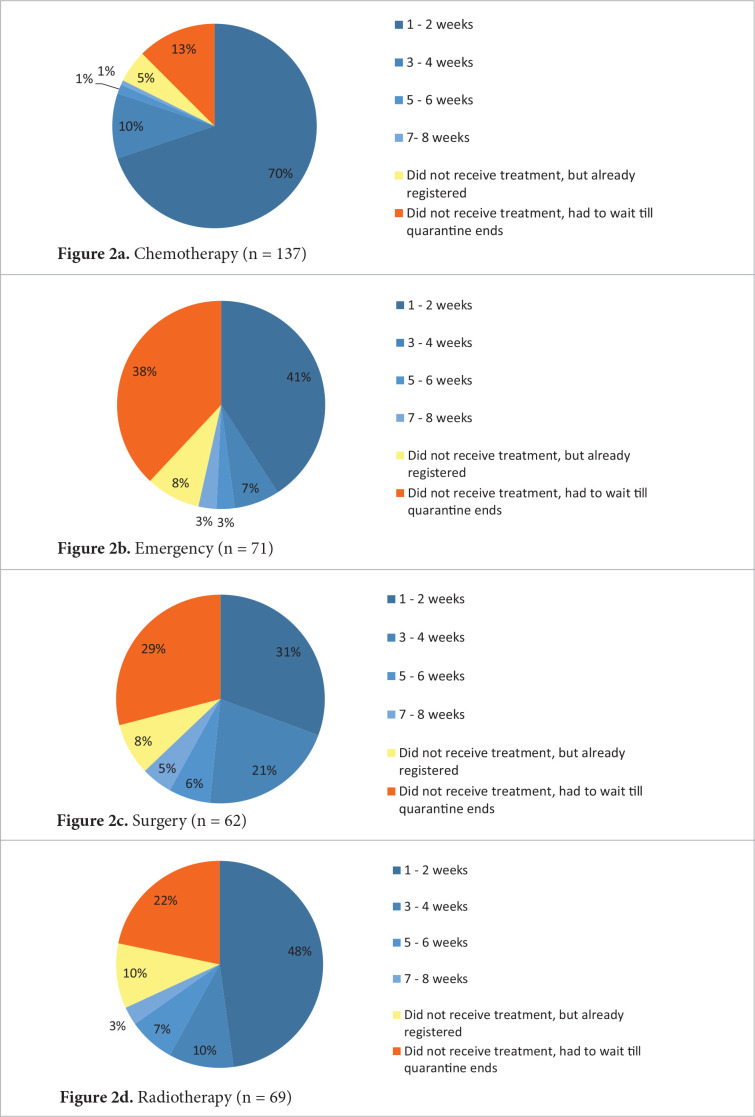
Cancer treatment during the quarantine

In recent guidelines, it is emphasized that diagnosis should not be compromised during COVID-19 pandemic; only the management of such patients should be tailored to the best available resources [5]. If specific treatment was initiated, 30% of patients did not receive consultation either. Chemotherapy, radiotherapy and surgery were also postponed until the pandemic subsides for 12.5, 21.7 and 29% of patients, respectively (Figure 2). In emergencies (quick worsening of symptoms) only 53.3% of respondents received help in 8 weeks. Some diagnostic tests were also not performed on time – about a third of patients had to wait until the end of quarantine for radiological, endoscopic or blood tests to be performed. 

The first wave of the COVID-19 pandemic is well controlled in Lithuania with only 1808 cases positive (66.4/100000), 1501 recovered and 78 deaths (in the time of writing this paper). This means that healthcare system should adapt quite well to the current situation because there is no overload. Perhaps strict policy regarding outpatient visits or hospital admissions made more damage than the COVID-19 pandemic itself. 

Other countries across Europe also reported cancer care services diminished dramatically, including UK, Italy, Germany and others [6]. When the first wave of COVID-19 subsides, hospitals will begin to continue their normal service, however, it seems that cancer patients will need some encouragement from healthcare professionals to come and not be afraid to contact the virus [6].

There are already some guidelines published online to help going through the healthcare system crisis [5,7–9]. They are usually based only on expert’s opinion, however, it is best what we have today. First, routine screening should be suspended and patients with early or advanced cancer be treated as outpatients as much as possible without referring to the main centres [7]. This crisis enables the use of telemedicine without contacting the patient directly. The focus should be on disease history and symptoms, however, even physical examination can be performed virtually. This approach during the COVID-19 pandemic is especially sufficient for surveillance [8]. The treatment for the first time diagnosed cancer can be initiated only after careful assessment of patient’s performance status, comorbidities, biology of disease and likely impact on quality of life if the treatment is initiated [7]. There are few types of cancer where we can expect good outcomes with treatment and the risk of delayed care is much more hazardous than the COVID-19 itself. This includes most patients with acute leukaemia, high-grade lymphoma, testicular, ovarian and small cell lung cancer [9]. Some oncologists see the COVID-19 pandemic not only as a global crisis, but also as an opportunity for clinical research, which would seem unethical under normal conditions [10]. Hypofractionated regimens for patients with limited additional benefit of regular regimens can be prescribed. In addition, neoadjuvant chemoradiotherapy instead of primary surgery can be initiated more freely by the multidisciplinary team. Chemotherapy doses can also be reduced to cause less immunosuppression [10]. On the other hand, all the ongoing preclinical trials should be stopped, unless there is a great early success with tumour-specific trial for which inclusion continues [10].

In conclusion, Lithuanian experience during the COVID-19 pandemic shows that the virus transmission can be successfully stopped. However, the price is paid on the account of patients with chronic diseases including cancer because of diminished access to medical care. About a third of patients did not receive any medical care during the quarantine. The balance between the risk of being infected with SARS-CoV-2 and the damage of delayed diagnosis and treatment of cancer can be achieved only with clear communication in the healthcare system and education of cancer patients and healthcare professionals. 
